# Schwangerschaftsabbrüche und Lebenslagen: Erkenntnisse auf Basis von *pairfam*-Daten

**DOI:** 10.1007/s00103-024-03997-0

**Published:** 2024-12-23

**Authors:** Lara Minkus

**Affiliations:** Seminar für Sozialstrukturanalyse, empirische Methoden und Statistik (SAMS), Europa-Universität Flensburg, Auf dem Campus 1, 24943 Flensburg, Deutschland

**Keywords:** Schwangerschaftsabbruch, Lebensverlaufsforschung, Fertilität, *pairfam*, Abortion, Life course research, Fertility, *pairfam*

## Abstract

**Hintergrund:**

Obwohl Schwangerschaftsabbrüche ein gesellschaftlich und politisch bedeutsames Thema darstellen, gibt es in Deutschland bisher kaum quantitative Untersuchungen zu den individuellen Lebensumständen der Betroffenen. Dieser Beitrag beleuchtet empirisch und deskriptiv die individuellen, ökonomischen und partnerschaftlichen Bedingungen, unter denen solche Entscheidungen getroffen werden.

**Methoden:**

Die Auswertungen basieren auf den Daten der ersten 13 Befragungswellen (2008–2021) des bundesweit durchgeführten Beziehungs- und Familienpanels (*pairfam*). Angaben von Frauen, die einen Schwangerschaftsabbruch vornehmen lassen, wurden mit jenen, die ihre Schwangerschaft austragen, hinsichtlich individueller Merkmale sowie ökonomischer und partnerschaftlicher Lebenslagen verglichen. Die Stichprobe besteht aus 1511 Beobachtungen von 1082 Befragten, darin enthalten sind 216 Schwangerschaftsabbrüche und 1295 Geburten. Die Daten wurden mittels deskriptiver Analyse ausgewertet.

**Ergebnisse:**

Schwangerschaftsabbrüche stehen mit verschiedenen individuellen Merkmalen sowie der ökonomischen und partnerschaftlichen Situation im Zusammenhang. Sie erfolgen vergleichsweise seltener bei guter Gesundheit und häufiger, wenn bereits 2 oder mehr Kinder vorhanden sind. Zudem ist die finanzielle Situation von Personen, die ihre Schwangerschaft abbrechen, vergleichsweise schlechter. Sie sind außerdem häufiger Singles oder mit ihrer Partnerschaft unzufrieden.

**Diskussion:**

Die Ergebnisse legen nahe, dass die Entscheidung für einen Schwangerschaftsabbruch im Kontext spezifischer Lebensumstände getroffen wird. Der vorliegende Beitrag unterstreicht die Notwendigkeit weiterer Forschung zu den komplexen Zusammenhängen.

## Abstract (en)

**Introduction:**

Despite the social and political importance of abortion, little quantitative research has been done on the individual circumstances of women who have had abortions in Germany. This article takes an empirical and descriptive look at the individual, economic, and partnership conditions under which such decisions are made.

**Methods:**

Data from the first 13 waves (2008–2021) of the German Family Panel (*pairfam*) were analyzed. Data from women who had an abortion were compared with those who carried the pregnancy to term in terms of individual characteristics, economic, and partnership circumstances. The sample consists of 1511 observations from 1082 respondents, including 216 abortions and 1295 births. The data were analyzed by means of descriptive analysis.

**Results:**

Abortions are associated with several individual, economic, and partnership characteristics. Abortions are relatively less frequent when women are in good health and more frequent when they already have two or more children. In addition, the financial situation of women who have an abortion is comparatively worse. They are also more likely to be single or dissatisfied with their partnership.

**Discussion:**

The findings suggest that the decision to terminate a pregnancy by abortion is made in the context of specific life circumstances. This article highlights the need for further research into the complexities involved.

## Hintergrund

Schwangerschaftsabbrüche nehmen in der öffentlichen Debatte großen Raum ein. Derzeit steht beispielsweise erneut die Strafbarkeit von Abbrüchen zur Diskussion, die bisher im Strafgesetzbuch in § 218 ff. geregelt ist. Eine von der Bundesregierung eingesetzte Expert*innengruppe hat jüngst empfohlen, die grundsätzliche Strafbarkeit des Abbruchs, zumindest bis zur 12. Woche, abzuschaffen. Die Kontroverse der Positionen zeigt sich aber nicht nur im Rahmen der Diskurse zu rechtlichen Regelungen des Schwangerschaftsabbruchs, sondern auch in konkreten gesellschaftlichen Auseinandersetzungen. Zu nennen ist hier beispielsweise die sogenannte Gehsteigbelästigung, also die Belästigung Schwangerer vor Beratungsstellen, Kliniken und Praxen durch Abtreibungsgegner*innen, die seit November 2024 mit einem Bußgeld geahndet wird. Trotz des breiten gesellschaftlichen und öffentlichen Interesses an diesem Thema ist die sozialwissenschaftliche Forschungslage insbesondere in Deutschland jedoch unzureichend [1].

Ein Forschungsdesiderat stellen Entscheidungsprozesse und sozioökonomische Lebenslagen von Frauen^1^ im Zusammenhang mit Schwangerschaftsabbrüchen dar [2]. Während sich einige internationale Studien durchaus quantitativ mit sozioökonomischen Lebenslagen und Motiven bei Schwangerschaftsabbrüchen beschäftigen (vgl. z. B. [3] und [4]), gibt es nach Kenntnis der Autorin in Deutschland nur wenige neuere quantitative empirische Studien zu diesem Thema. Eine dieser wenigen Studien, die ein umfassendes und detailliertes Bild der Lebenslagen ungewollt Schwangerer zeichnet, basiert zwar auf einer Zufallsstichprobe, diese ist aber regional begrenzt [5]. Eine weitere ist im Hinblick auf gängige empirische Gütekriterien als problematisch einzustufen, unter anderem ist hier zu bemängeln, dass die Studie auf einer selektiven und nicht probabilistischen Stichprobe beruht [6].^2^ Lediglich eine Studie [2], die sich mit individuellen Lebenslagen und deren Zusammenhang mit Schwangerschaftsabbrüchen beschäftigt, verwendet Paneldaten, die auf einer bundesweiten Kohortenzufallsstichprobe basieren.

Die Frage nach dem Zusammenhang zwischen individueller Lebenssituation und Schwangerschaftsabbruch stellt sich auch, weil Schwangeren, die sich für einen Abbruch entscheiden, häufig eine gewisse Impulsivität unterstellt wird. Dies zeigt sich unter anderem in der verpflichtenden 3‑tägigen Wartezeit, die zwischen einer Schwangerschaftskonfliktberatung und dem tatsächlichen Abbruch liegen muss, um einen Abbruch nach der Beratungsregel straffrei durchführen zu können. Auf der Internetseite des Bundesministeriums der Justiz (BMJ) ist dazu zu lesen, dass diese 3‑Tages-Frist dazu dienen soll, „überstürzte Entscheidungen“ [8] zu verhindern.

Der vorliegende Beitrag hat das Ziel, die skizzierte Forschungslücke zumindest teilweise zu schließen. Anhand von Daten des Beziehungs- und Familienpanels (*pairfam*) werden deskriptive Einblicke in die Lebenssituation von Personen gegeben, die sich für einen Schwangerschaftsabbruch entscheiden. Die vorliegende Arbeit soll damit auch einen möglichen Bezugsrahmen für zukünftige Forschung schaffen. Dabei gliedert sich der Beitrag zunächst in eine kurze Darstellung des Schwangerschaftsabbruchs und seiner rechtlichen Rahmenbedingungen in Deutschland, gefolgt von theoretischen Überlegungen und einer Beschreibung der verwendeten Daten und Methoden. Der Beitrag schließt mit den Ergebnissen der empirischen Analyse und einer Diskussion.

### Schwangerschaftsabbrüche in Deutschland

Im länderübergreifenden Vergleich gibt es in Deutschland vergleichsweise wenig Schwangerschaftsabbrüche, wobei die Abbruchraten in den östlichen Bundesländern höher sind als in den westlichen [9]. Der Großteil der Abbrüche in Deutschland erfolgt nach der Beratungsregelung und findet innerhalb der ersten 12 Wochen statt [10]. Schwangere Personen, die einen Abbruch vornehmen lassen, sind überwiegend zwischen 25 und 34 Jahre alt [10]. Zudem ist die Zahl der Schwangerschaftsabbrüche in Deutschland in den letzten Jahrzehnten rückläufig [10]. Die Zahlen des Statistischen Bundesamtes zeigen auch für die Pandemiezeit in den Jahren 2020 und insbesondere 2021 einen Abwärtstrend. Neuere Zahlen zu Schwangerschaftsabbrüchen aus den Jahren 2022 und 2023 zeigen hingegen einen Aufwärtstrend. Dies deutet darauf hin, dass die vergleichsweise niedrigeren Abbruchraten in den Jahren 2020 und insbesondere 2021 vermutlich auch auf einen pandemiebedingt erschwerten Zugang zu Beratung und medizinischer Versorgung zurückzuführen sind [11]. Umfassende wissenschaftliche Untersuchungen stehen jedoch noch aus.

Schwangerschaftsabbrüche sind im Strafgesetzbuch geregelt und damit grundsätzlich strafbar. Sie sind jedoch rechtmäßig und straffrei, wenn es sich um eine medizinische Notwendigkeit handelt oder wenn die Schwangerschaft durch eine Vergewaltigung herbeigeführt wurde (kriminelle Indikation). Sie sind ebenfalls straffrei, wenn die betreffende Person weniger als 12 Wochen schwanger ist und mindestens 3 Tage vor dem Abbruch an einer Schwangerschaftskonfliktberatung in einer anerkannten Beratungsstelle teilgenommen hat [8]. Ein Schwangerschaftsabbruch kostet in Deutschland in der Regel zwischen 300 € und 600 €, die medizinische Vor- und Nachsorge wird von den gesetzlichen Krankenkassen übernommen [12]. Liegt das monatliche Einkommen der Person, die einen Schwangerschaftsabbruch vornehmen lassen möchte, unter einer bestimmten Grenze, werden die Kosten des Eingriffs auf Antrag bei der Krankenkasse übernommen. Die Kosten werden ebenfalls übernommen, wenn die Schwangerschaft für die schwangere Person ein gesundheitliches Risiko darstellt oder im Zusammenhang mit einer Sexualstraftat entstanden ist, also nach der Indikationsregel abgebrochen wird [12].

### Theoretische Überlegungen zu Fertilität und Schwangerschaftsabbruch

Ein großer Teil der Fertilitätsforschung beschäftigt sich mit den Fertilitätsintentionen. Dabei wird untersucht, warum und unter welchen Umständen sich Individuen und Paare für Kinder entscheiden. Für Teile des globalen Nordens hat sich gezeigt, dass es eine Lücke zwischen der gewünschten und der tatsächlichen Kinderzahl gibt. Diese Lücke ist negativ, d. h., es werden weniger Kinder geboren als gewünscht [13]. Empirische Arbeiten befassen sich daher zumeist mit den Gründen, warum Individuen und Paare Kinder bekommen, oder eben den Gründen, warum die realisierte Kinderzahl geringer ist als die gewünschte. Weniger gut erforscht ist hingegen die Kehrseite, nämlich warum sich Schwangere und Paare gegen Kinder entscheiden.

Angelehnt an Huinink und Kohli [14] gehen Minkus und Drobnič [13] davon aus, dass die Entscheidung für einen Schwangerschaftsabbruch von den Ressourcen der Betroffenen, ihren vergangenen und gegenwärtigen Lebenslagen und -umständen beeinflusst wird. Dem Lebensverlaufsansatz folgend, werden Familiengründung und Elternschaft als instrumentelle Ziele der individuellen Wohlfahrtsproduktion im Lebensverlauf betrachtet, mit der Konsequenz, dass die Entscheidung für ein Kind nur dann getroffen wird, wenn es zur eigenen Wohlfahrt beiträgt. Umgekehrt schlagen die Autorinnen vor, dass bestimmte Lebensumstände systematisch die Wahrscheinlichkeit beeinflussen, dass sich Frauen oder Paare für einen Schwangerschaftsabbruch entscheiden [2]. Die Entscheidung für oder gegen eine Schwangerschaft wird also, angelehnt an bisherige Forschung, als geplante Entscheidung betrachtet [15]. Diese Entscheidung ist eingebettet in sich verändernde ökonomische und sozialstrukturelle Bedingungen, die den Kinderwunsch und damit auch die Entscheidung zum Schwangerschaftsabbruch beeinflussen.

Im Folgenden wird untersucht, inwieweit individuelle Lebenslagen mit dem Auftreten von Schwangerschaftsabbrüchen zusammenhängen. Im Einzelnen werden folgende Teilbereiche näher betrachtet: individuelle Merkmale und ökonomische Situation sowie die Art und Qualität der Partnerschaft. Die empirische Umsetzung erfolgt mittels deskriptiver Statistik.

## Methoden

### Daten

Die vorliegende Studie verwendet Daten des Release 14.1 des Beziehungs- und Familienpanels *pairfam *(„Panel Analysis of Intimate Relationships and Family Dynamics“) aus den Jahren 2008–2021 (Welle 1–13; [16]). Die Daten zu partnerschaftlichen und familialen Lebensformen in Deutschland wurden bis 2022 jährlich auf Basis einer bundesweiten, zufallsbasierten Kohortenstichprobe erhoben [17].

### Variablen

Schwangerschaftsabbrüche sind für die Betroffenen in der Regel ein sensibles Thema. Daher besteht bei der Erfassung von Schwangerschaftsabbrüchen im Rahmen von Befragungen das Problem, dass die Antworten durch soziale Erwünschtheit verzerrt sein können [3, 18]. Um den Einfluss sozialer Erwünschtheit zu minimieren, werden im *pairfam* die Fragen zum Schwangerschaftsabbruch mittels CASI (computergestütztes Selbstinterview) erhoben.^3^ Bei dieser Erhebungsmethode bedienen die Befragten den von dem oder der Interviewer*in zur Verfügung gestellten Computer selbst, ohne dass Dritte ihre Antworten einsehen können. Empirische Evidenz deutet darauf hin, dass die Untererfassung von Schwangerschaftsabbrüchen bei computergestützter Selbstbefragung erheblich reduziert wird [19]. Darüber hinaus haben die Befragten die Möglichkeit, auch die Antwortkategorie „Ich möchte das nicht beantworten“ auszuwählen und haben somit die Option, das Frageitem zu beantworten, ohne sensible Informationen preiszugeben oder falsche Angaben zu machen. Zusammengenommen ist davon auszugehen, dass diese Maßnahmen eine mögliche Verzerrung des Antwortverhaltens durch soziale Erwünschtheit reduzieren.

Eine methodische Schwierigkeit besteht darin, aus den Daten den genauen Zeitpunkt des Schwangerschaftsabbruchs zu bestimmen. Die Frageformulierung für weibliche Befragte lautet: „Haben Sie seit der letzten Befragung einen Schwangerschaftsabbruch vornehmen lassen?“, das heißt es liegt nur die Information vor, dass zwischen dem letzten Interview (t-1) und dem aktuellen Interview (t) ein Schwangerschaftsabbruch stattgefunden hat, der genaue Zeitpunkt ist jedoch unbekannt. Um dennoch die aus der Datengenese logische zeitliche Abfolge zu berücksichtigen, wurde die Variable zum Schwangerschaftsabbruch jeweils aus der aktuellen Welle (t) entnommen, während die meisten Variablen zu individuellen Lebensumständen aus der Befragung stammen, die ein Jahr vor den Angaben zu Schwangerschaftsabbrüchen (t-1) stattfand.

Lebenslagen wurden zunächst anhand individueller Merkmale operationalisiert. Neben dem Alter in Kategorien (< 20 Jahre, 20–25 Jahre, 26–29 Jahre, 30–34 Jahre, 35–40 Jahre und > 40 Jahre), ob und wie oft eine Religionsstätte besucht wird sowie der subjektiven Einschätzung des eigenen Gesundheitszustandes (sehr schlecht/schlecht, zufriedenstellend oder sehr gut/gut) wurde auch die Anzahl der leiblichen Kinder in Kategorien (keine Kinder, ein Kind, 2 oder mehr Kinder) berücksichtigt.

Zur Operationalisierung der individuellen ökonomischen Situation wurden subjektive Geldsorgen sowie die Zufriedenheit mit den Haushaltsfinanzen herangezogen. Letztere wurde auf einer Skala von 0 („sehr unzufrieden“) bis 10 („sehr zufrieden“) erhoben und für die folgenden Berechnungen in 3 Quantile unterteilt. Darüber hinaus wurde zur Messung der wirtschaftlichen Situation eine mögliche Arbeitslosigkeit von mindestens einem Monat berücksichtigt. Diese Variable wurde mithilfe des Event-History-Kalenders in *pairfam* erhoben und umfasst den Zeitraum zwischen der letzten (t-1) und der aktuellen Befragungswelle (t).

Darüber hinaus wurden Partnerschaftsmerkmale untersucht. Analysiert wurden der Beziehungsstatus sowie die Zufriedenheit mit der beruflichen und häuslichen Arbeitsteilung. Außerdem wurde die Beziehungszufriedenheit, gemessen auf einer Skala von 0 („sehr unzufrieden“) bis 10 („sehr zufrieden“) und für die vorliegenden Analysen in 3 Quantile unterteilt, untersucht. Weiterhin wurde mithilfe des Event-History-Kalenders erhoben, ob es zwischen der letzten und der aktuellen Welle zu einer Trennung von dem oder der Partner*in kam.

### Stichprobe

Die Ausgangsstichprobe, bestehend aus allen *pairfam-*Basisbefragten,^4^ enthält 80.010 Beobachtungen, welche wiederum von 12.402 Befragten stammen. Zunächst wurden alle Personen, die sich nicht als weiblich identifizierten, aus der Stichprobe entfernt, was 37.692 Beobachtungen ausmachte. Um den Personen, die sich gegen das Austragen der Schwangerschaft entschieden hatten, eine aussagekräftige Vergleichsgruppe gegenüberzustellen, wurden die Personen, die ihre Schwangerschaft abgebrochen haben, nicht nur innerhalb ihrer eigenen Gruppe verglichen, sondern auch mit sich als weiblich identifizierende Befragte, die zwischen der letzten (t-1) und der aktuellen Befragung (t) ein Kind bekommen haben. Daher wurden alle Beobachtungen entfernt, bei denen zwischen der aktuellen und der letzten Welle weder ein Schwangerschaftsabbruch noch eine Geburt vermerkt wurde. Die Zahl der Beobachtungen verringert sich dadurch um 40.557 Fälle. Schließlich wurden alle Beobachtungen gelöscht, bei denen das letzte Interview mehr als ein Jahr zurücklag, was 127 Beobachtungen entspricht. Außerdem wurden 108 Beobachtung aufgrund fehlender Werte auf der Schwangerschaftsabbruchvariablen gelöscht. Schließlich wurden 15 Fälle ausgeschlossen, für die in den Daten sowohl eine Geburt als auch ein Schwangerschaftsabbruch im letzten Jahr vermerkt war, da dies zwar möglich, aber eher unwahrscheinlich ist.

Die endgültige Stichprobe enthält damit 1511 Beobachtungen von 1082 Befragten, bestehend aus 216 Abbrüche und 1295 Geburten. Da die Fallzahlen der folgenden deskriptiven Analysen aufgrund unterschiedlicher Anzahl fehlender Werte auf den verschiedenen Variablen variieren, ist in jeder Abbildung auch die Anzahl der gültigen Beobachtungen („*n*“) angegeben.

### Analysen und Darstellung

Die im Folgenden dargestellten Analysen basieren auf deskriptiven Berechnungen, die mithilfe von Balkendiagrammen dargestellt werden. Die Abbildungen geben jeweils Auskunft über den Zusammenhang zwischen Schwangerschaftsabbrüchen und Geburten und den oben genannten individuellen, ökonomischen und partnerschaftlichen Indikatoren. Alle Auswertungen wurden mit den von *pairfam* bereitgestellten kalibrierten Designgewichten für die Basisstichprobe (cdweight) gewichtet [21]. Die Abbildungen wurden mithilfe von Stata 17 erstellt.

## Ergebnisse

**Abb. 1:**
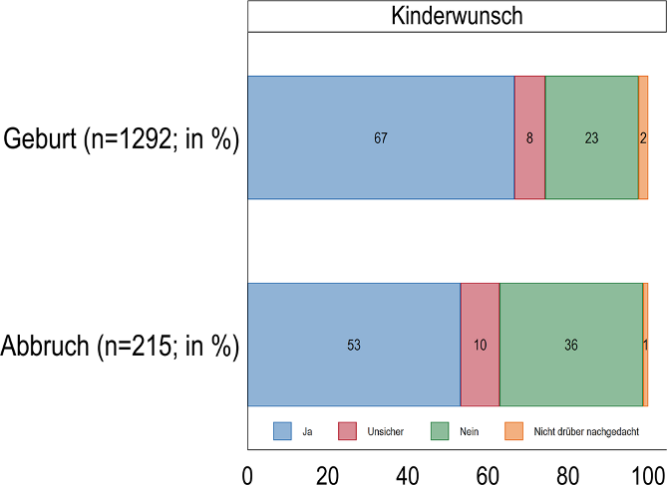
Kinderwunsch von Frauen, die ihre Schwangerschaft austragen (Geburt) oder abbrechen (Abbruch). Relative Anteile der Antwortkategorien (%). (Quelle: *pairfam* Datenrelease 14.1 (2008–2021), eigene Berechnungen, gewichtet)

Nicht nur 67 % der Frauen, die ihre Schwangerschaft austragen, gaben einen Kinderwunsch an, sondern auch 53 % der Frauen, die sich für einen Schwangerschaftsabbruch entscheiden (Abb. 1). Weitere 10 % der Frauen, die ihre Schwangerschaft abbrechen, sind sich unsicher, ob sie (noch) ein Kind wünschen, und 36 % schließen (weitere) Kinder aus. Dies kann damit als Hinweis verstanden werden, dass die Entscheidung für oder gegen einen Schwangerschaftsabbruch in einigen Fällen nicht prädeterminiert ist, sondern sich verändernden individuellen Lebensumständen unterliegt. Dieser Befund deckt sich mit empirischer Evidenz zur Variabilität von Fertilitätsintentionen: Auch der Kinderwunsch ist im Lebensverlauf keineswegs stabil, sondern wird ebenso von sich verändernden Lebensumständen beeinflusst [22, 23]. Welche Lebenslagen statistisch gehäuft im Zusammenhang mit Schwangerschaftsabbrüchen auftreten, wird in den folgenden deskriptiven Analysen dargestellt.

### Individuelle Merkmale

**Abb. 2:**
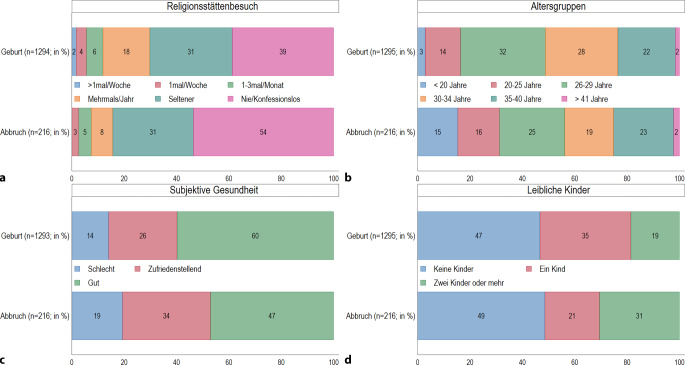
**a**–**d** Individuelle Merkmale von Frauen, die ihre Schwangerschaft austragen (Geburt) oder abbrechen (Abbruch). Relative Anteile der Antwortkategorien (%). (Quelle: *pairfam *Datenrelease 14.1 (2008–2021), eigene Berechnungen, gewichtet)

Fast 61 % der Personen, die ihre Schwangerschaft austragen, suchen zumindest hin und wieder eine Religionsstätte auf; dies trifft nur auf 47 % derer zu, die ihre Schwangerschaft abbrechen (Abb. 2a).^5^ Darüber hinaus machen die Analysen deutlich, dass die Abbruchquoten bei den jüngeren Befragten vergleichsweise hoch sind (Abb. 2b): Zwar sind nur 3 % der Personen, die ihre Schwangerschaft austragen, zum Zeitpunkt der Befragung unter 20 Jahre alt, jedoch betreffen 15 % aller Schwangerschaftsabbrüche die unter 20-Jährigen. Bei den 26- bis 29-Jährigen kehrt sich das Bild um: In dieser Altersgruppe tragen 32 % ihre Schwangerschaft aus, während 25 % der 26- bis 29-Jährigen ihre Schwangerschaft abbrechen. Diese Tendenz zeigt sich auch unter den 30- bis 34-Jährigen. Bei den 20- bis 25-Jährigen und den 35- bis 40-Jährigen sind die Anteile in beiden Gruppen relativ ausgeglichen.

Frauen, die ihre Schwangerschaft abbrechen, geben häufiger einen schlechten oder zufriedenstellenden Gesundheitszustand an (53 %) als Personen, die ihre Schwangerschaft austragen (40 %; Abb. 2c). Von den Frauen mit Schwangerschaftsabbruch geben 49 % an, keine leiblichen Kinder zu haben (Abb. 2d). Dieser Anteil ist nur geringfügig höher als der Anteil der bisher kinderlosen bzw. kinderfreien Frauen, die sich für eine Geburt entscheiden (47 %). Gleichwohl werden 31 % der Schwangerschaftsabbrüche von Frauen vorgenommen, die bereits 2 oder mehr Kinder haben. Zum Vergleich: Von den Frauen, die ihre Schwangerschaft austragen, haben nur 19 % bereits 2 oder mehr Kinder.

### Ökonomische Situation

**Abb. 3:**
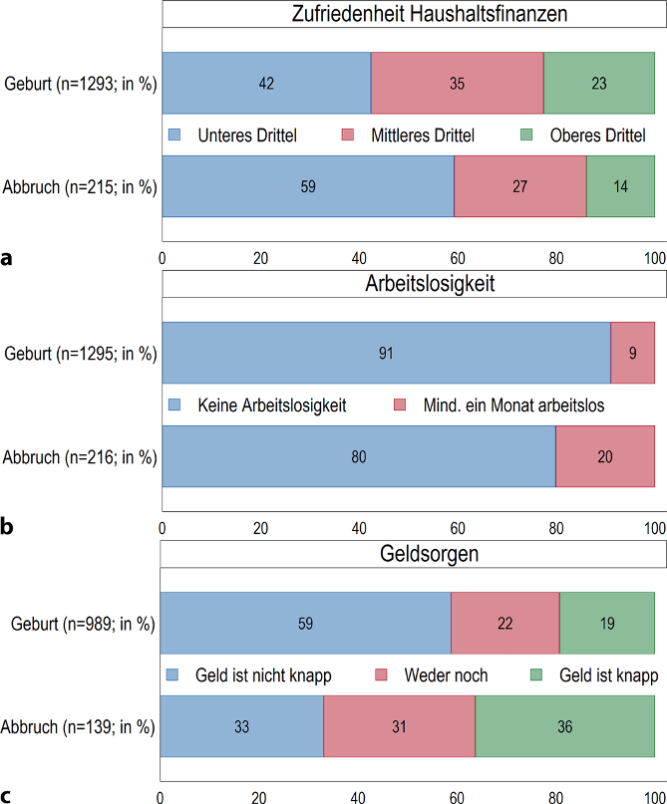
**a**–**c** Ökonomische Situation von Frauen, die ihre Schwangerschaft austragen (Geburt) oder abbrechen (Abbruch). Relative Anteile der Antwortkategorien (%). (Quelle: *pairfam* Datenrelease 14.1 (2008–2021), eigene Berechnungen, gewichtet)

Es zeigt sich ein deutlicher Zusammenhang zwischen der finanziellen Situation und der Entscheidung für oder gegen das Austragen einer Schwangerschaft: 59 % der Frauen, die ihre Schwangerschaft abbrechen, befinden sich im unteren Drittel der Zufriedenheit mit der finanziellen Situation des Haushalts (Abb. 3a). Dies trifft nur auf 42 % der Frauen zu, die sich für ein Austragen entscheiden. 20 % der Frauen, die ihre Schwangerschaft abbrechen, sind mindestens einen Monat arbeitslos. Bei den Frauen, die die Schwangerschaft austragen, sind es nur 9 % (Abb. 3b). Knapp bei Kasse sind 36 % der Frauen, die ihre Schwangerschaft abbrechen, und 19 % der Frauen, die ihre Schwangerschaft austragen (Abb. 3c).

### Partnerschaft

**Abb. 4:**
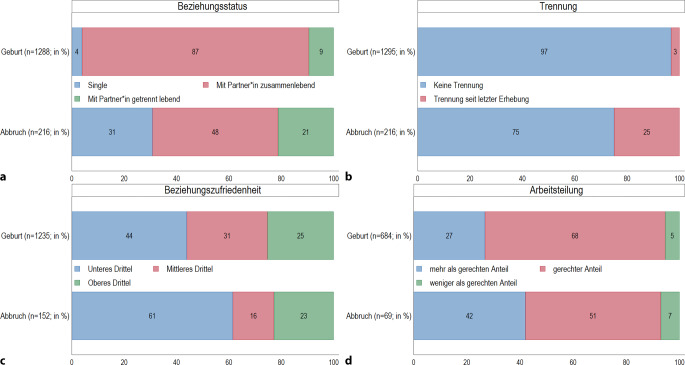
**a**–**d** Partnerschaftssituation von Frauen, die ihre Schwangerschaft austragen (Geburt) oder abbrechen (Abbruch). Relative Anteile der Antwortkategorien (%). (Quelle: *pairfam* Datenrelease 14.1 (2008–2021), eigene Berechnungen, gewichtet)

Von den Frauen, die ihre Schwangerschaft abbrechen, leben 31 % als Single und 21 % in einer festen Beziehung, aber von ihrem Partner getrennt (Abb. 4a). Demgegenüber lebt ein Großteil (87 %) der Personen, die ihre Schwangerschaft austragen, in einer Partnerschaft mit gemeinsamem Haushalt. 25 % der Personen, die ihre Schwangerschaft abbrechen, haben sich innerhalb des letzten Jahres von ihrem Partner oder ihrer Partnerin getrennt (Abb. 4b). Das trifft nur auf 3 % derjenigen zu, die ihre Schwangerschaft austragen.

Die Beziehungszufriedenheit befindet sich bei 61 % der Personen, die ihre Schwangerschaft abbrechen, im unteren Drittel (Abb. 4c). 42 % der Schwangeren, die sich für einen Schwangerschaftsabbruch entscheiden, empfinden die Arbeitslast in der Partnerschaft als ungerecht zu ihren Ungunsten verteilt, während dies nur auf 27 % der Befragten zutrifft, die ihre Schwangerschaft austragen (Abb. 4d).

## Diskussion

Der vorliegende Beitrag beschäftigt sich mit dem Zusammenhang von individuellen, ökonomischen und partnerschaftlichen Lebenslagen und Schwangerschaftsabbrüchen. Die Ergebnisse zeigen, dass Frauen, die sich für einen Schwangerschaftsabbruch entscheiden, vergleichsweise oft bei schlechter Gesundheit sind und häufiger 2 oder mehr Kindern haben. Zudem haben sie häufig finanzielle Sorgen. Im Vergleich zu den Frauen, die sich für das Austragen der Schwangerschaft entscheiden, schätzen sie ihre Partnerschaft hinsichtlich der Beziehungsqualität häufig als schlechter ein. Außerdem haben sie vergleichsweise häufig eine Trennung erlebt und empfinden ihren Anteil an der partnerschaftlichen Arbeitslast als ungerecht. Die Ergebnisse verdeutlichen, dass die Entscheidung für einen Abbruch nicht isoliert zu betrachten ist, sondern eingebettet ist in Partnerschaftsstrukturen, ökonomische und individuelle Lebenslagen, die es zu berücksichtigen gilt.

Die Ergebnisse dieser Analysen sind aufgrund ihres deskriptiven Charakters als Anregung für weitere Forschungen zu verstehen. Die Analysen beschränken sich auf eine reine Beschreibung mit dem Ziel, Forschungspotenziale in einem bisher wenig bearbeiteten Forschungsfeld aufzuzeigen. Eine Quantifizierung der Stärke der dargestellten Zusammenhänge ist nicht das Ziel dieser Arbeit. Ebenso wenig sollen und können kausale Zusammenhänge erklärt werden. Die hier vorgestellten deskriptiven Analysen reichen nicht aus, um die komplexen Zusammenhänge zwischen Lebenslagen und Schwangerschaftsabbrüchen in ihrer Vielschichtigkeit zu erfassen. Der vorliegende Beitrag gibt jedoch Hinweise darauf, in welcher Richtungen sich weitere Untersuchungen lohnen.

Für diese weiterführende Forschung wäre es allerdings dringend notwendig, die bereits erwähnte spärliche Datenlage zu Schwangerschaftsabbrüchen in Deutschland zu verbessern. Die lückenhafte Datenlage in Verbindung mit dem großen öffentlichen Interesse begünstigt die Veröffentlichung von Studien mit Daten, die wissenschaftlichen Qualitätskriterien nicht genügen (siehe [6] und ausführlich dazu auch Fußnote 2). Die zu erwartenden Ergebnisse der ELSA-Studie sowie der aktuell laufenden Befragung „frauen leben 4“ sind sicherlich wertvolle Meilensteine auf dem Weg zu einer dichteren Datenlage.

## Fazit

Zusammenfassend konnte in dieser Arbeit gezeigt werden, dass eine Vielzahl von Lebensumständen mit der Entscheidung für einen Schwangerschaftsabbruch zusammenhängen. Weitere Studien und eine verbesserte Datenlage sind notwendig, um die verschiedenen Faktoren zu identifizieren und entsprechend wirksame, auf die individuellen Lebensumstände abgestimmte Unterstützungsmaßnahmen zu entwickeln.

## Footnotes

1 Nicht nur Frauen können schwanger werden, dieser gesellschaftlichen Realität soll dadurch Rechnung getragen werden, dass in diesem Text häufig auch von schwangeren Personen die Rede ist. In den Analysen werden jedoch nur Personen berücksichtigt, die sich selbst als weiblich identifizieren, sodass im Folgenden häufig auch nur von Frauen gesprochen wird.

2 Diese Studie basiert unter anderem auf Daten aus Gedächtnisprotokollen des Beratungstelefons VITaL. VITaL wird von der Aktion Lebensrecht für Alle (ALfA e. V.) betrieben. Die ALfA schreibt auf ihrer Website [7]: „[…] Abtreibungen können daher weder erlaubt noch gesellschaftlich akzeptiert werden und sind schon gar nicht Bestandteil des Frauenrechts.“ Diese Datenbasis ist also weder im umgangssprachlichen Sinne „repräsentativ“, noch erfüllt sie die übrigen Gütekriterien quantitativer Forschung. Die Ergebnisse dieser Studie und weiterer Studien des Autors, die auf der gleichen Datenbasis beruhen, sind daher mit Vorbehalt zu betrachten.

3 Ab Welle 12 wurde die Befragung aufgrund der COVID-19-Pandemie nicht nur im CAPI- (Computer Assisted Personal Interviewing) respektive CASI-Modus, sondern auch telefonisch (CATI) und durch die Befragten selbst im Paper and Pencil Interview (PAPI) durchgeführt. In den telefonischen Interviews sowie im PAPI-Fragebogen der Welle 13 und in der gesamten Welle 14 wurde die Frage zum Schwangerschaftsabbruch nicht erhoben.

4 Für eine Übersicht zu den in *pairfam *gezogenen Stichproben siehe [20].

5 In einer multivariaten Analyse, in die auch Personen einbezogen wurden, die in der Befragung keine Angaben zu einem möglichen Schwangerschaftsabbruch machen wollten, konnte dieser Zusammenhang jedoch nicht bestätigt werden (siehe dazu [2]). Soziale Erwünschtheit im Antwortverhalten bei Befragungen zum Schwangerschaftsabbruch scheint also bei Personen, die in eine religiöse Gemeinschaft eingebunden sind, eher eine Rolle zu spielen, in dem Sinne, dass sie in Befragungen weniger offen zugeben, wenn sie einen Schwangerschaftsabbruch vorgenommen haben. Hier besteht jedoch weiterer Forschungsbedarf.
